# Otitis Externa in a Newborn After Waterbirth—A Case Report Followed by a Literature Review

**DOI:** 10.3390/jcm15166490

**Published:** 2026-08-21

**Authors:** Julia Ufnal, Maria Wolniewicz, Lidia Zawadzka-Głos

**Affiliations:** 1 Students’ Scientific Association of Pediatric Otorhinolaryngology “Otorhino”, University Department of Pediatric Otolaryngology, Medical University of Warsaw, Zwirki I Wigury 63A, 02-091 Warsaw, Poland; 2 University Department of Pediatric Otolaryngology, Medical University of Warsaw, Zwirki I Wigury 63A, 02-091m Warsaw, Poland

**Keywords:** waterbirth, waterbirth complications, water delivery, acute otitis externa, neonates, neonatal external ear infection, post-delivery care

## Abstract

Waterbirth has become a widely adopted alternative to a conventional delivery, with well-documented maternal benefits, but there is an ongoing debate regarding neonatal safety. We would like to report a case of a 10-day-old male neonate who developed acute otitis externa (AOE) following waterbirth. Despite the rare occurrence of this disease in children under 2 years of age, prompt diagnosis and timely initiation of appropriate therapy led to a full recovery. This case represents a previously undocumented, yet plausible, complication of waterbirth and underscores the importance of careful neonatal ear assessment and preventive measures in aquatic deliveries.

## 1. Introduction

Waterbirth, defined as labor and/or delivery occurring in a birthing pool or tub filled with warm water, has gained increasing popularity over recent decades as an alternative to a conventional delivery. Originating as a form of hydrotherapy in the 18th century and inspired by the practices of ancient Egyptian, Roman, and Greek physicians [1], it has evolved into a recognized obstetric practice in several healthcare systems, including the British National Health Service, where it is even incorporated into official guidelines [2]. Its reported maternal benefits include pain relief, shorter labor duration, reduced need for epidural anesthesia, increased endorphin levels, decreased catecholamine release, improved uterine perfusion, and greater maternal satisfaction [3,4]. Some systematic reviews also suggest improved neonatal outcomes, including fewer NICU admissions and a lower incidence of low Apgar scores [4]. The safety of waterbirth remains debated, particularly regarding the risk of neonatal infections following water immersion during birth. Although most studies have not demonstrated higher infection rates compared to the land births, isolated reports of severe complications, including *Pseudomonas aeruginosa* sepsis, *Legionella pneumophila* infection, and group B streptococcal meningitis, underscore the need for vigilance [5,6]. The unique environment of the birthing pool, which can be warm, humid, and potentially contaminated, may theoretically provide favorable local conditions for pathogens’ growth leading to various infections. Below, we present a case of a newborn who contracted otitis externa after waterbirth, was consequently hospitalized in the university pediatric hospital, and was consulted by the otorhinolaryngological department, with the latter leading to the final diagnosis.

## 2. Case Report

A 10-day-old male neonate was admitted to the university pediatric hospital with a 3-day history of whitish discharge from the left ear accompanied by irritability, especially when placed or cuddled on the left side, and poor feeding, as reported by the mother. The boy was born at 38 weeks of gestation via waterbirth (all phases of labor with water delivery) as the first child of a 29-year-old woman (gravida 1, para 1), with no history of maternal illnesses. The infant was exclusively breastfed and received the recommended neonatal vaccinations. On admission, the boy was in good general condition, afebrile, and hemodynamically stable. Physical examination revealed whitish discharge in the left external auditory canal. A swab was taken for microbiological testing, and then it was cleaned with suction. The skin of the external auditory canal was erythematous and swollen, resulting in concentric stenosis of its lumen and limited visualization of the tympanic membrane. Marked tenderness of the tragus and auditory ear canal (when using speculum or other instruments) was also noted. However, despite the stenosis, visualized fragment of the tympanic membrane appeared pale and translucent, excluding otitis media, cholesteatoma or temporal bone tumors involving middle or external ear structures. The post-auricular area and auricula were not erythematous or swollen, with no signs of the auricula protrusion. The right ear examination was unremarkable. Both pediatric and otolaryngological examinations showed no signs of a systemic infection. The final diagnosis of an acute otitis externa (AOE) was established by the otolaryngologist. The initial diagnosis made by the pediatric team was acute otitis media (AOM) with tympanic membrane perforation, as visualization of the ear was limited before precise cleaning. Therefore, they insisted on systemic antibiotic therapy with intravenous ampicillin. At the same time, daily antiseptic cleaning of the external auditory canal with boric acid and topical ciprofloxacin administration were performed, according to the otorhinolaryngological consultation. On the third day of hospitalization, microbiological culture results demonstrated growth of *Pseudomonas aeruginosa* cultures, sensitive to ciprofloxacin—a typical pathogen responsible for otitis externa. Due to the local improvement, systemic antibiotic therapy was ceased, and the child was discharged home with topical ciprofloxacin drops continued until the seventh day, together with strict avoidance of water exposure. At the follow-up visit one week later, according to the mother’s report, the child remained asymptomatic, with no complaints or signs of a recurrent infection. Otoscopic examination revealed satisfactory healing of the left external auditory canal, showing pale skin covered with administered drops, absence of edema, and a translucent tympanic membrane without signs of an acute infection.

## 3. Discussion

For women who meet specific criteria, immersion hydrotherapy represents a viable birth option to consider and might be discussed during the antenatal period to ensure conscious decision-making. This method may be applicable in low-risk pregnancies over 37 gestational weeks, with a singleton fetus in cephalic presentation, stable maternal and fetal status, blood pressure within the reference range, and clear amniotic fluid. Contraindications include high maternal BMI (body mass index), intrauterine growth restriction, antepartum hemorrhage, herpes simplex infection, or unstable fetal heart rate [7].

The maternal benefits of this delivery method are well-documented and often cited by its advocates. In addition to the aforementioned pain relief, reduced use of analgesics, shorter labor duration, and greater maternal satisfaction are also benefits [3]. It has also been proven that waterbirths have lower intervention rates. Immersion in warm water increases the blood flow and the temperature within the muscles and joints, leading to muscle relaxation, which in turn positively affects the uterine blood flow and oxytocin secretion. As a result, the pelvic diameter expands and contractions are more effective, the vaginal tissues stretch more easily, and the elasticity of the perineum increases [8]. Simultaneously, the oxygen and blood supply to the fetus increase as well [9]. Warm water also facilitates maternal–infant bonding and promotes skin-to-skin contact, as well as helps in body temperature regulation [9]. For women meeting specific eligibility criteria, such as gestational age over 37 weeks, cephalic presentation, and absence of comorbidities, waterbirth has also been linked to another significant benefit—lower cesarean section rates compared to national averages [10]. Women declare that they most often choose waterbirth for pain relief, a sense of natural and empowering birth, the calming environment it provides, personal preference or based on recommendations from other women [1].

Although the advantages for the mother are well-documented, neonatal outcomes are still uncertain. Published studies have demonstrated a reduction in both the first and the second stages of labor, and a lower incidence of neonatal facial hematocyanosis, when compared with conventional deliveries [11]. On the other hand, researchers reported a greater prevalence of umbilical cord detachment in waterbirths compared with the land births [12]. Newborns delivered by this method are also at risk of respiratory distress due to birthing pool water aspiration [13]. It is worth mentioning that the lower incidence of low Apgar scores observed by Cluett et al. [4] may be due to the analysis bias, as only low-risk pregnant women are eligible for water birth. Another study found no difference in Apgar scores between the compared delivery methods [11]. While immersion during the first stage of labor is generally considered as low risk, immersion during the second stage, particularly during the exact moment of delivery, remains more controversial due to possible neonatal risks, most notably infection and aspiration [14].

Szymkowiak et al. did not observe any infections among 210 newborns delivered in water. They suggested that in an aquatic environment, the amniotic membrane ruptures later due to a smaller pressure gradient, which potentially protects the child from probable infections [11]. While most studies have not found higher infection rates when compared with the land births [12,15,16], rare, but severe, complications have been documented in the literature, including *Pseudomonas aeruginosa* sepsis, *Legionella pneumophila* pneumonia and group B streptococcal meningitis [5,13,17]. Water quality, temperature, and hygiene measures are critical in minimizing such risks [6]. The warm, humid environment of birthing pools may serve as a reservoir for opportunistic pathogens. To reduce the risk of infection, waterbirth protocols recommend using only regular tap water without additives, such as essential oils, maintaining the water temperature below 37.5 °C, ensuring complete immersion of the perineum, and responding quickly to the contamination by replacing or filtering the water. Women are advised to leave the pool to urinate, and facilities should be prepared for an immediate evacuation, if necessary [10]. An interesting prospective study by Thoni et al. proved that the installment of filter system significantly reduced the presence of *Legionella* from 29% to 0%, and *Pseudomonas aeruginosa* from 22% to 3% in samples taken just after filling the tube with tap water [16]. This is an important aspect that should be considered by centers offering waterbirths. On the contrary, samples taken after the water delivery were contaminated, not surprisingly, by colibacilli in 81% of samples and Escherichia coli in 58%; these rates were not linked with the increase neonate infection rate requiring antibiotics, and were even smaller than in conventionally born neonates (2.30% vs. 1.15%) [16]. No colibacilli or Escherischia coli were found in the first sample, so this may be the proof for a low risk of a cross-infection.

Moreover, waterbirth may alter early intestinal bacterial colonization and thereby affect the establishment of the gut microbiome. Given the critical role of the microbiome in immune system maturation, metabolic regulation, and disease susceptibility, such alterations may impact general health not only in the neonatal period, but also later in adulthood [6]. In a similar manner, it may also interfere with bacterial skin colonization by washing out fetal vernix and biofilm formation, with this interference being widely documented as a risk factor for AOE [18].

An interesting aspect to consider is also meconium-stained amniotic fluid (MSAF) and its potential role in predisposing for AOE. MSAF, also referred to as green-stained amniotic fluid, is a condition when an in utero defecation is observed [19]. This may be either a physiological phenomenon or the result of hypoxia, intraamniotic infection, or complication of post-term pregnancies [19]. Unlike the intestinal content of adults and children that are rich in bacteria, meconium during fetal life is sterile [19]. MSAF is a risk factor for neonatal hypoxic–ischemic encephalopathy, neonatal sepsis, neonatal seizures, meconium aspiration syndrome (MAS), and cerebral palsy [19]. We found no published articles that linked this condition with AOE; however, due to a relatively low risk of AOE compared with other complications, this may be often omitted. We believe that this problem needs further investigation.

AOE is a frequent condition in the pediatric population, and comprises a diffuse inflammation of the external auditory canal. It predominantly affects children over the age of two, with the highest incidence observed in those aged 5 to 14 years, particularly during the summer months [6,20,21]. Approximately 90% of cases are unilateral [20]. Commonly known as *swimmer’s ear*, it is typically associated with a history of prolonged water exposure [21]. Risk factors also include humidity, skin macerations, mechanical trauma, the use of external devices (e.g., headphones, hearing aids, earplugs), dermatological disorders, anatomical abnormalities, a compromised immune system, and obstruction of the auditory canal (earwax plug, foreign body, etc.) [20,21,22]. The most common mechanism involves moisture exposure, which leads to maceration of the ear canal epithelium and elevation of its pH. However, all the above-mentioned risk factors may damage the epithelial barrier, thereby promoting local infection. The most frequently identified etiological pathogens are *Staphylococcus aureus* and *Pseudomonas aeruginosa* [20,21,23]. The diagnosis of AOE is based on characteristic clinical symptoms and signs with a rapid onset, usually within 48 h [20]. It obviously requires differentiation from other potential causes of ear pain. In neonates, otitis externa may clinically resemble AOM [24]. These two conditions can be distinguished by the presence and/or character of the external auditory canal edema (concentric in AOE, absent in classic AOM and asymmetric, with prolapse of the posterior-upper wall of the external auditory canal in complicated AOM—mastoiditis) as well as the nature of a discharge—purulent without mucus, a positive tragal tenderness sign and appearance of the tympanic membrane (typically normal in AOE). However, in extreme cases, it may be barely distinguishable between those two conditions—postauricular inflammatory reaction vs. mastoiditis—and a CT scan is required for the final verification. Although relatively rare, fungal otitis externa should also be taken into consideration. It may be characterized by a creamy exudate in the case of *Candida albicans* infection or a black discharge in *Aspergillus niger* infection; sometimes fungal hyphae may also be observed [24]. In doubtful or recurrent cases, microbiological culture results support the final diagnosis and may influence the choice of therapeutic approach. In neonates, it is also important to consider the possibility that debris in the external auditory canal may represent milk precipitate regurgitated from the mouth, which collects in the concha and external auditory canal—a condition referred to as “milk-induced otitis externa”. Affected infants typically present with whitish ear discharge and mild ear tenderness, while the tympanic membrane remains normal. In such cases, cultures of the discharge are negative [24]. What may also hinder the right diagnosis in newborns is their naturally narrow external auditory canal, requiring adjusted instruments (e.g., speculums, suction devices) and experience.

Literature addressing AOE in the neonatal population is limited. Sadeghi et al. reported a case of AOE in a 29-day-old, vaginally delivered, born-on-time, otherwise healthy neonate who presented with swelling, erythema and edema of the left auricle, irritability, and poor feeding [22]. Unusually for this diagnosis, the infant also developed a diffuse erythematous macular rash on the trunk, which resolved after treatment. Another report described a 23-day-old girl, the first-born of twins, who presented with irritability and discharge from the right ear [25]. The chest X-ray was normal, while blood and urine cultures were negative. In both cases, ear discharge cultures revealed *Staphylococcus aureus*. Intravenous antibiotic therapy was initiated and subsequently switched to oral antibiotics after discharge, leading to complete recovery. Follow-up visits showed no signs of complications or recurrence.

The treatment of uncomplicated AOE generally involves topical antibiotics, thorough cleaning, and avoidance of water exposure. Topical treatment in the form of ear drops is recommended as the first-line treatment, as it provides the highest drug concentrations—even 100 to 1000 times greater than during systemic therapy. At the same time, it minimizes systemic side effects and the risk of antibiotic resistance induction [20]. More severe cases should be managed with systemic antibiotics that cover *Streptococcus aureus* and *Pseudomonas aeruginosa*. Clinical improvement is typically observed within 48 to 72 h; however, a complete response may take up to six days in advanced cases [26]. Systemic antibiotics are not recommended as the initial treatment for uncomplicated AOE according to the American Academy of Otolaryngology, Head and Neck Surgery Foundation guidelines, though these recommendations do not explicitly include neonates or children younger than two years [27]. It is also worth mentioning that the choice of oral antibiotics in young patients is especially restricted, as fluoroquinolones known for their antipseudomonal activity are contraindicated in this population due to their potential risk of growth cartilage damage [28]. Therefore, cases refractory to topical treatment usually require intravenous treatment. Systemic therapy should be based on bacterial culture and sensitivity, although empiric antibiotics that target *Pseudomonas aeruginosa* (e.g., ceftazidime) are appropriate [29].

As noted by Jyai Allen et al., conducting a randomized trial directly comparing waterbirth with non-waterbirth is unlikely to be feasible [30]. It is difficult to identify a similar low-risk cohort of women for comparison or to randomly decide on the method of birth. This makes case reports an important source of data on rare and potentially underrecognized complications.

In our case, the onset of symptoms within days after birth, absence of other infection sources, and documented exposure to birthing water suggest a plausible link between waterbirth and the development of otitis externa. Although rare, this association highlights a need to consider external ear hygiene as part of neonatal assessment following aquatic delivery. The immature skin barrier of newborns, combined with possible subclinical abrasions during labor, may facilitate microbial invasion even in the absence of systemic illness. The assumption stated in the case report is highly possible in our opinion; however, as a limitation of our study, as we did not perform day-by-day examination of the child since birth and some hygienic procedures after birth could have been done, together with usually non-invasive neonatal screening, we cannot prove it with 100% assurance. It is essential that women are clearly informed about the potential risks and benefits prior to immersion to ensure autonomous, evidence-based choices in pain relief and birth planning.

## 4. Conclusions

Due to the exposure to the aquatic environment, high temperatures, and potential mechanical trauma, clinicians should consider otitis externa as a potential complication of waterbirths. Increased awareness may facilitate a prompt diagnosis of an infection if it occurs in a newborn and assist parents in making more informed decisions regarding the optimal delivery method. Preventive strategies may include thorough post-delivery ear drying, rigorous monitoring of water quality, and educating birth attendants to recognize subtle otologic signs. Neonatal care protocols may benefit from incorporating routine external ear inspection in the postnatal assessment of infants delivered in water. So far, no published case report has established a direct association between neonatal otitis externa and waterbirth. This rare clinical occurrence opens a discussion regarding the need for greater awareness of subtle and potentially underreported complications related to immersion in water deliveries. Moreover, it highlights the importance of an early recognition and treatment of otologic infections in the neonatal population.

## Data Availability

The data presented in this study are available on request from the corresponding author.

## Abbreviations

AOE	Acute otitis externa
AOM	Acute otitis media
BMI	Body mass index
MAS	Meconium aspiration syndrome
MSAF	Meconium-stained amniotic fluid
NICU	Neonatal intensive care unit
